# Valve Type and Operative Risks in Surgical Explantation of Transcatheter Aortic Valves: A Systematic Review and Meta-Analysis

**DOI:** 10.3390/jcm13051262

**Published:** 2024-02-23

**Authors:** Riccardo G. Abbasciano, Dimitrios E. Magouliotis, Marinos Koulouroudias, Kyriakos Spiliopoulos, Andrew Xanthopoulos, Antonios Kourliouros, Roberto Casula, Thanos Athanasiou, Alessandro Viviano

**Affiliations:** 1 Department of Cardiothoracic Surgery, Imperial College Healthcare NHS Trust, London W12 0HS, UK; riccardo.abbasciano@nhs.net (R.G.A.); roberto.casula@nhs.net (R.C.); 2 Department of Surgery & Cancer, Imperial College, London SW7 2BX, UK; t.athanasiou@imperial.ac.uk; 3 Unit of Quality Improvement, Department of Cardiothoracic Surgery, University of Thessaly, Biopolis, 41 110 Larissa, Greece; dimitrios.magouliotis.18@alumni.ucl.ac.uk; 4 Department of Cardiac Surgery, Nottingham University Hospitals NHS Trust, Nottingham NG7 2UH, UK; marinos.koulouroudias1@nhs.net; 5 Department of Cardiothoracic Surgery, University of Thessaly, Biopolis, 41 110 Larissa, Greece; spiliopoulos@med.uth.gr; 6 Department of Cardiology, University Hospital of Larissa, 413 34 Larissa, Greece; andrewvxanth@gmail.com; 7 Department of Cardiothoracic Surgery, John Radcliffe Hospital, Oxford University Hospitals NHS Foundation Trust, Oxford OX3 9DU, UK

**Keywords:** TAVR, systematic review, surgical explantation of TAVR

## Abstract

Indication to perform surgical explantation of TAVR is becoming increasingly more frequent, due to the higher number of transcatheter procedures performed in patients with longer life expectancy. We proposed to perform a systematic review and meta-analysis with metaregression to identify potential factors that can determine an increase in the high mortality and morbidity that characterize these surgical procedures. MEDLINE and Embase were searched for relevant studies. Twelve studies were eligible according to our inclusion criteria. TAVR explantation was confirmed as a procedure with high 30-day mortality (0.17; 95% CI, 0.14–0.21) and morbidity (stroke incidence 5%; 95% CI, 0.04–0.07; kidney injury incidence 16%; 95% CI, 0.11–0.24). The type of transcatheter valve implanted during the index procedure did not influence the outcomes after surgical explantation. The role of these high-risk operations is growing, and it will likely expand in the coming years. Specific tools for risk stratification are required.

## 1. Introduction

Transcatheter aortic valve replacement (TAVR) has provided a minimally invasive alternative to surgical aortic valve replacement (SAVR) for the treatment of severe aortic stenosis, gaining rapid traction due to its reduced invasiveness, shorter hospital stays, and decreased perioperative morbidity compared to traditional surgical interventions. Recent advancements in valve design, delivery systems, and patient selection criteria have led to the expansion of indication to carry out TAVR, leading to a broader patient population being treated, not exclusively those deemed high-risk or inoperable for SAVR.

Studies have shown the clinical efficacy and safety of TAVR across various patient cohorts. The PARTNER (Placement of Aortic Transcatheter Valves) trial [1] demonstrated the non-inferiority of TAVR compared to SAVR in high-risk patients, and subsequent trials, such as the PARTNER 2 [2] and the Evolut Low-Risk trials [3], extended these findings to intermediate and low-risk populations, respectively. The PARTNER 3 trial [4] reported a lower incidence of a composite outcome of death, stroke, or rehospitalization at 1 year with TAVR than compared to SAVR, although with a follow-up of 1 year.

**Figure 1:**
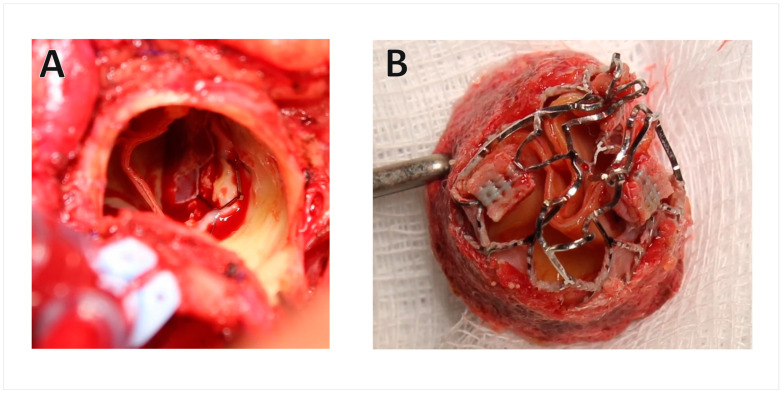
**Example of indication to perform TAVR explantation.** TAVR explant performed in a patient that had been implanted 1 year earlier with Sapien 3 Ultra 23 (requiring chimney stent during the TAVI for left main stem coronary protection) for structural valve degeneration of surgical aortic bioprosthesis (Abbott Trifecta size 23), after evidence of chimney stent stenosis and HALT of the transcatheter valve. (**A**) Intraoperative photo. (**B**) Explanted TAVR. *AVR—aortic valve replacement; HALT—hypo-attenuation leaflet thickening; TAVR—transcatheter aortic valve replacement*.

The degeneration of transcatheter aortic valves (TAVs) represents a crucial clinical concern in the field of interventional cardiology and transcatheter aortic valve replacement. A growing body of research has underscored the potential for TAV degeneration, which can manifest as structural valve deterioration, valve thrombosis, or paravalvular leak. Structural valve deterioration involves the progressive deterioration of valve leaflets and can lead to valve stenosis or regurgitation. Recent studies have pointed to the multifactorial nature of TAV degeneration [5,6,7], implicating factors such as biomechanical stress, inflammation, and calcification. Valve thrombosis, characterized by the formation of thrombotic material on valve leaflets, has been linked to impaired valve function and has been studied extensively [8] (Figure 1). Paravalvular leak, the presence of perivalvular gaps allowing retrograde blood flow, is another significant concern [5], as it can contribute to haemodynamic compromise and increased mortality. Lastly, endocarditis is a rare but serious complication after TAVI, with an incidence ranging from 0.2% to 3.1% at 1 year post implant, associated with high mortality and morbidity [9]. The most common causative organisms are Enterococci, Staphylococcus aureus, and coagulase negative staphylococci. Risk factors include younger age, male sex, valve design, and increasing post-deployment peak gradient. The management endocarditis after TAVI is challenging, as transcatheter procedures are precluded by the infectious process in most cases.

The long-term implications of TAVI degeneration are still being elucidated, but they constitute a clinical problem of growing importance, in particular in view of the frequent lack of transcatheter treatments available for these patients [10]. The presence of endocarditis, unfavourable anatomy (due to the risk of coronary ostia obstruction or to the size of the annulus), and need for concomitant procedures are frequent contraindications to TAVI-in-TAVI [10], and while relatively low, the rate of surgery for TAVR explant is increasing, likely due to the growing number of younger patients who have undergone these procedures in recent years. Hospital mortality for these operations remains high [11].

We aimed to update the work by Yokoyama et al. [11] by reviewing the current scientific literature regarding TAVR explant to identify potential explanatory features that could contribute to the technical complexity of this surgery (type of transcatheter valve implanted, indication for explant) to understand whether these could affect mortality and morbidity. Our secondary aim was to explore the published literature to define procedural strategies for aortic repair in case of complex surgical scenarios (damage to the aortic wall, small-size root).

## 2. Materials and Methods

A literature review was performed to identify relevant studies that explored the topic of surgical explant of transcatheter aortic valves. The study protocol was registered prospectively on PROSPERO (CRD42023477435). The PRISMA checklist is available in the Supplementary Materials.

Potentially eligible studies were identified by searching electronic databases including MEDLINE and EMBASE, using a combination of subject headings and text words to identify relevant studies. Searches were not restricted by language or publication status. The reference lists of eligible studies and reviews also were examined. Searches were last updated on the 10 August 2023.

Details of publications, design, and key characteristics of the research work assessed were extracted from eligible studies onto an electronic data collection database.

Two authors (RGA and AV) screened the search output independently to identify records of potentially eligible studies. After exclusion of studies that were clearly not relevant following a review of study titles and abstracts, the full texts of eligible studies were obtained and assessed for inclusion. Discrepancies were resolved through consensus and consultation with a third investigator (TA). A standardized form was used for data extraction and included year of publication, country of participants recruited, participant demographic features, and nature of treatment, along with indications for treatment and baseline condition, and for likely sources of bias. The data were recorded and subsequently tabulated with Microsoft Excel (Microsoft Corp, Redmond, WA, USA).

Dichotomous data were reported as risk ratios (RR) with 95% confidence intervals (CIs) and continuous data as standardized mean difference (SMD) with 95% CIs. The analytic approach to the unit of analysis issues, and missing data, was as described in the Cochrane Handbook [12]. The I^2^ statistic was used to measure heterogeneity for each outcome among the studies in each analysis. Metaregression (redo status, endocarditis as an indication, and self-expandable/balloon-expandable patients in the cohort) analysis, using a mixed-effects model, was performed for moderator exploration. Analyses were carried out using the R software package 4.3.2 (R Foundation for Statistical Computing, Vienna, Austria).

A narrative descriptive synthesis was conducted when no quantitative analysis could be performed.

The risk of bias assessment tool described by Hoy et al. was used to rate the methodological quality of the studies [13].

## 3. Results

### 3.1. Search Results

**Figure 2:**
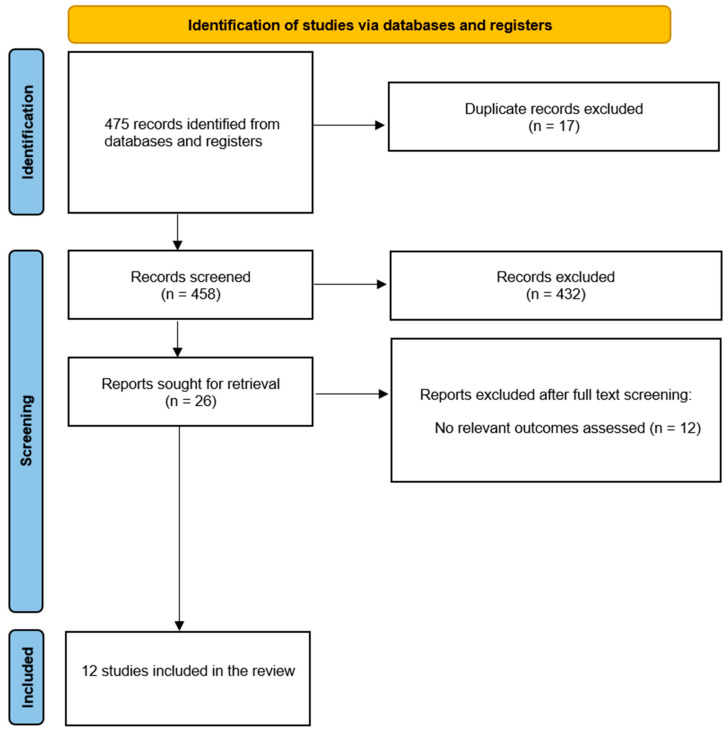
**PRISMA 2020 flow diagram for the studies included in the review.** PRISMA 2020 flow diagram describing the identification, screening, and inclusion phases of the studies in the review.

A total of 475 abstracts were retrieved from the searches (Figure 2). After removal of duplicate entries, 459 articles were screened, of which 432 were excluded on the basis of titles and abstracts; a total of 26 relevant publications were retrieved for further assessment. Twelve studies [14,15,16,17,18,19,20,21,22,23,24,25], analysing a total of 1584 participants receiving TAVR explant, met the inclusion criteria and were included in the quantitative analysis. A summary of the characteristics of the studies included is reported in Table 1. There was no disagreement among the reviewers as to the selection of the studies. A summary of the judgements regarding potential sources of bias in the studies is presented in Table 2.

### 3.2. Characteristics of Included Studies

The studies included cover a period from 2008 to 2020. More than 300,000 patients underwent TAVI in the cohort examined, with an average requirement for surgical explant of 0.6%, occurring after a median of 247 days (IQR 228–360) from the index procedure. The average risk at explant, calculated with STS-PROM, was 9.75%, as expected higher than the risk at implant, 5.42%. Thirty-seven percent of the participants in the studies had undergone previous cardiac surgery. The number of balloon-expandable valves removed was 626, while the number of self-expandable valves removed was 342. The most frequent mechanisms of valve failure were endocarditis (403 total cases, an average of 43.9% of incidence in the series reported), structural valve deterioration (320), aortic regurgitation (266), and aortic stenosis (171). A failure in implantation occurred in 279 cases (an average of 10.4% of incidence in the series reported). An isolated surgical aortic valve replacement was sufficient in 793 patients (an average of 48% among the series reported). Concomitant procedures to repair the aorta were required in 344 cases (an average of 25.8% among the series reported), more frequently in the region of the root (256 patients, average of 17.4%) than in the ascending aorta (159 cases, 13.8%).

### 3.3. Bias Assessment

The overall quality of the retrieved evidence was moderate. The series included assessed cohorts that were close to a true representation of the target population (which is different from the national one, as expected). The likelihood of nonresponse bias was minimal, and outcome assessment (definitions and methods of collection) was adequate. A short follow-up was common among the included studies and could have probably introduced bias.

### 3.4. Main Analysis

**Figure 3:**
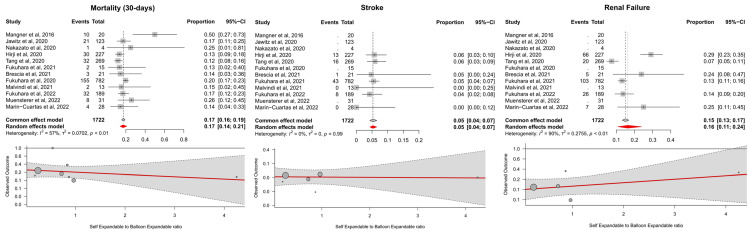
**Summary of the main results from the systematic review.** Forest plots tables for the outcomes of mortality, stroke, and renal failure, with metaregression plots below exploring the relationship between these outcomes and the ratio between self-expandable and balloon-expandable valves in the studies [14,15,16,17,18,19,20,21,22,23,24,25]. *CI—confidence interval*.

The forest plots with pooled estimates from random-effects meta-analyses are reported in Figure 3. In the primary analysis, 30-day mortality among the cohorts was 17% (12 studies; proportion 0.17; 95% CI, 0.14–0.21; I^2^ = 57%) with moderate heterogeneity across the studies, while the incidence of stroke was 5% (seven studies; proportion 0.05; 95% CI, 0.04–0.07; I^2^ = 0%). The proportion of patients suffering periprocedural kidney injury was 16% (seven studies; proportion 0.16; 95% CI, 0.11–0.24; I^2^ = 90%), but significant heterogeneity could be appreciated across the studies.

A metaregression was performed to investigate the role of the ratio between self-expandable valves and balloon-expandable valves as a moderator on the primary outcomes. The type of transcatheter devices used during the index procedures did not significantly affect the incidence of mortality (*p* = 0.493), stroke (*p* = 0.941), or renal injury (*p* = 0.333).

### 3.5. Secondary Analyses

**Figure 4:**
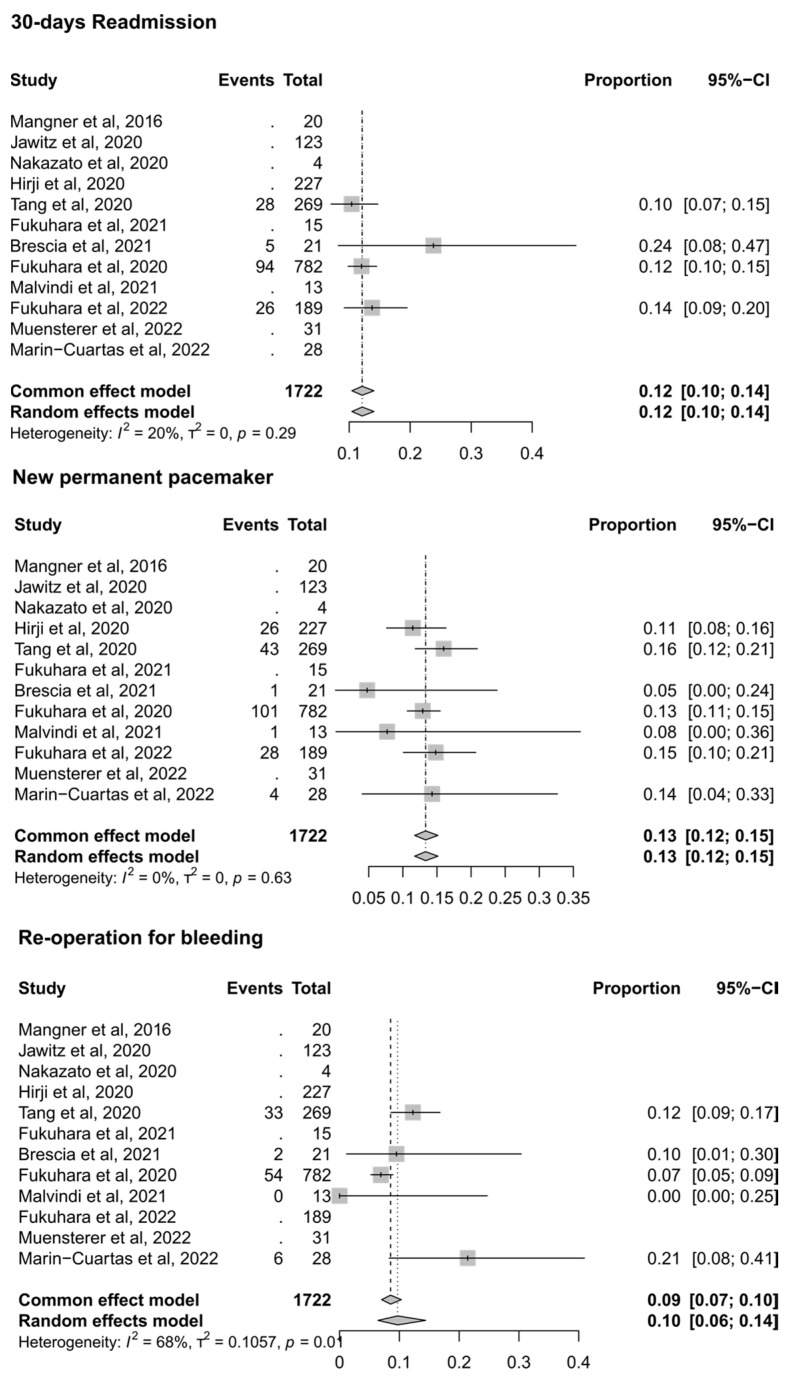
**Forest plots for secondary outcomes.** Forest plots for the outcomes of 30-day readmission, new permanent pacemaker, and re-operation for bleeding [14,15,16,17,18,19,20,21,22,23,24,25]. *CI—confidence interval*.

Additional analyses were performed for secondary outcomes (Figure 4). Readmissions at 30 days were 12% (four studies; proportion 0.12; 95% CI, 0.10–0.14; I^2^ = 20%). A need for a new permanent pacemaker insertion occurred in 13% of cases (seven studies; proportion 0.13; 95% CI, 0.12–0.15; I^2^ = 0%). A re-operation for bleeding was required in 10% of the cases (five studies; proportion 0.10; 95% CI, 0.06–0.14; I^2^ = 68%); moderate heterogeneity could be appreciated across the studies reporting this outcome.

The type of transcatheter valves at index procedure did not influence 30-day readmission rates (*p* = 0.306) or the need for pacemaker implant (*p* = 0.429), re-operation for bleeding (*p* = 0.681), or the need for a concomitant aortic repair (*p* = 0.303).

The redo status of the procedure was explored as a potential moderator as it could add complexity to the surgery but was found not to significantly influence mortality (*p* = 0.582), stroke (*p* = 0.828), renal injury (*p* = 0.218), readmission rate (*p* = 0.829), permanent pacemaker implant (*p* = 0.955), or re-operation rate (*p* = 0.074).

Similarly, endocarditis as an indication to perform surgery did not account for heterogeneity significantly in all of the outcomes, mortality (*p* = 0.110), stroke (*p* = 0.597), renal injury (*p* = 0.937), readmission rate (*p* = 0.574), permanent pacemaker implant (*p* = 0.715), or re-operation rate (*p* = 0.109).

### 3.6. Narrative Synthesis of Procedural Considerations

Heterogeneity in reporting and a lack of robust quantitative data reporting prevented us from conducting a meta-analytical pooling of procedural considerations. Nonetheless, the following considerations could be summarized from a qualitative review of the available evidence.

The work by Vitanova et al. [26] provides insights into the comparison between aortic valve and aortic root replacement following TAVR explant, through their analysis of the EXPLANT-TAVR registry, which included patients who had their TAVR device explanted between November 2009 and September 2020. Patients who had their TAVR device explanted during the same hospitalization as their initial TAVR procedure were excluded; 168 patients received SAVR and 28 patients root replacement as their primary aortic valve intervention after TAVR explant. Among all TAVR explant procedures in the registry, root replacement was therefore performed exclusively in 14% of all cases (18.7% of self-expandable valves and 10.5% of balloon expandable valves). The patients who needed root replacement had less comorbidities but more anatomical features that made redo TAVR complex. They also had less urgent or emergency cases and a longer median time from their first TAVR procedure to their TAVR explant (17.6 vs. 9.9 months; *p* = 0.047). Patients who receive root replacement also had more concurrent procedures involving the ascending aorta (58.8% vs. 14.0%; *p* < 0.001). The median follow-up time after TAVR explant was 6.9 months (interquartile range, 1.4–21.6 months) and 97.4% complete. The survival rate at follow-up was 81.2% and did not differ between the two groups (logrank *p* = 0.54).

One possible factor that affects the difficulty of removing the transcatheter heart valve from the native valve and annulus is the duration between the initial TAVR and the subsequent TAVR explantation. The longer this interval is, the more likely it is that the THV will develop a layer of endothelial cells on its surface, which can adhere to the surrounding tissues and make the separation process more complex and risky. Nonetheless, in terms of the relation between time of surgery and risk, Brescia et al. [14] found no statistically significant difference between late vs. early explants (14% vs. 6.5%; *p* = 0.13).

Considerations about root enlargement indications and techniques are not specific to the TAVR explant population and can be inferred from the ones available in the wider surgical population of patients with small annuli (effective orifice area index below ≤0.85 cm^2^/m^2^, particularly in young patients). Aortic root enlargement is currently performed in 10% of cases [27] via different surgical methods such as the extension of the aortotomy posteriorly, either through the non-coronary sinus (Nicks procedure) or through the left/non-coronary commissure and the anterior mitral leaflet (Manouguian procedure), or the use of the Konno procedure (seldom performed in adult patients), which augments the anterior annulus and the right ventricle [28,29,30]. More recently, a root enlargement with a Y incision has been proposed [31]. Replacing the aortic root during SAVR can also prevent patient–prosthesis mismatch and is safe without extra risk in centres with adequate experience, but its long-term benefits are still uncertain [32].

In terms of valve choice, sutureless prostheses might offer better haemodynamics, but the literature on their use specifically after TAVR explant is lacking. Nonetheless, in the analysis of 59 patients who underwent redo SAVR with self-expandable sutureless aortic bioprosthesis in 17 international centres [33], the median stay in the intensive care unit was 2 days, and the survival rate at 5 years was 96.3%, with a mean pressure gradient across the valve at 1 year follow-up of 11.2 ± 5.2 mmHg, confirming how sutureless valves in redo surgery are a safe and effective option compared to traditional AVR techniques.

## 4. Discussion

The role of surgical explantation of TAVI valves will likely expand in the future, with the increase in the use of TAVI valves in younger cohorts, hence the importance of understanding optimal patient selection and how to improve the current results [34].

We reviewed the published outcomes of over 1500 patients undergoing TAVR explant. We encountered no differences relating to the type of TAVR implanted, or to the indication for the index procedure or at explant.

The available evidence should be included in the risk stratification and shared decision-making process and guide clinicians’ management of patients with aortic stenosis; a long-term strategy that looks beyond the first procedure, and to the need for potential reintervention in the future, should be implemented whenever appropriate. Contemporary data about surgical aortic valve replacement confirm safe perioperative outcomes and excellent survival at follow-up [35].

Despite its successes, several periprocedural complications and long-term issues are a cause of concern when extending the indication to carry out transcatheter treatment to cohorts with a long life expectancy [36]. Paravalvular leak, conduction abnormalities, vascular complications, and long-term durability and outcomes require investigation in younger and lower-risk patients [37]. The expanding use of TAVR, encompassing an increasingly diverse patient population, highlights the need for robust data collection, multidisciplinary collaboration, and long-term surveillance to ensure its safety. In this setting, the emerging role of surgical explant and its high rate of complication and mortality is a reason to adopt caution in young patients considering TAVR implant. This is even more relevant as the cohort studies seemingly supporting TAVR-in-TAVR as a safe, widespread procedure [38] fail to provide a clear denominator that would allow a clear understanding of the number of patients for whom a repeat transcatheter procedure is unfeasible [39].

TAVR-in-TAVR is an option that presents several issues due to the technical complexity of the procedure, and it is not always a feasible strategy to treat patients with failing transcatheter valves. In fact, access to coronary ostia is frequently a contraindication that precludes a transcatheter approach, and techniques such as BASILICA [40] and chimney stenting are not always sufficient to prevent coronary obstructions. Moreover, haemodynamic parameters of TAVR valves in valves are still under investigation, and they may carry implications for TAVR durability [41], which need to be taken into consideration when planning redo TAVR procedures.

By updating the work by Yokoyama et al. [11], the present work confirms the high surgical risk reported for explanting transcatheter aortic valves, which is significantly higher than the one calculated at the index procedure, but more importantly, it is also increased compared to the figure expected by using current risk stratification tools. Moreover, at the present stage, a tool focused on this specific patient category is still unavailable. In our review, we investigated potential differences in outcomes between type of surgical explant required. Despite the potential added complexity due to the formation of neointima on the stent meshes [17], resulting in higher requirements of aortic procedures, explanting a self-expandable valve was not shown to have an increased risk in our work. Possibly, as the procedures of valve explantation are usually performed in aortic centres with a high level of technical skills, the complexity of the procedure has a minor role determining the outcomes compared to the baseline characteristics of the patients, their frequent multimorbidity and frailty, and the role that these factors play in the post-operative period.

Although we did not find a significant role of endocarditis as a moderator that increases the mortality or morbidity, surgical explantation of TAVI in this context is a challenging procedure that requires careful assessment of the patient’s condition and the extent of infection. TAVI valves are more prone to develop infective endocarditis in young patients and in the presence of increased transvalvular gradients [9,21]. The indications for surgery include valve dysfunction, persistent infection refractory to antibiotic therapy, peri-annular complications, and systemic embolization [21]. The surgical technique involves radical debridement of all infected and necrotic tissue, removal of the TAVI prosthesis (using different methods such as Kocher clamps, endarterectomy spatula, or heavy snaring sutures), and reconstruction of the aortic root with a bioprosthetic or mechanical valve. The outcomes of surgical treatment are poor, with high rates of mortality and morbidity [42].

Limitations of our work are related to the non-randomized and retrospective nature of most of the studies included in the meta-analysis, which reduces the certainty of evidence. Also, important factors such as the surgical access, anthropometric characteristics, and pharmacological treatments were not consistently recorded and could not be reliably investigated as moderators influencing the outcome.

As transcatheter valve devices become more diverse and widely used, cardiac surgeons will have to learn about their different features, how they vary, and how they might deteriorate over time (or acutely). Depending on the type of transcatheter device that was implanted and the patient’s specific condition and anatomy, different techniques of explantation may be required. Also, surgeons that approach this cohort of patients will require expertise in enlarging and replacing the aortic root, along with treating potential accompanying valvular and coronary disease [43].

## 5. Conclusions

TAVR explantation remains a procedure characterized by a high risk of mortality and morbidity, regardless of the indication to carry out the surgery itself. The type of valve implanted at the index procedure does not influence the outcome of the surgery. Further studies with longer follow up and larger cohorts are required to stratify the risk for the patients presenting with this emerging indication; the identification of different factors influencing the outcome should be considered a research priority, considering the unusually high ratio between observed and expected deaths when using current risk calculators.

## Figures and Tables

**Table 1:** Characteristics of included studies.

Study	Study Period	TAVR Valve Explant, *n*	Days from Implant (SD)	STS-PROM at Implant, %, Mean ± SD	STS-PROM at Explant, %, Mean ± SD	Balloon-Expandable Valve, *n*	Self-Expandable Valve, *n*	SVD, *n*	PVL/Aortic Insufficiency, *n*	Endocarditis, *n*	Failed Implantation, *n*	Concomitant Aortic Repair
**Brescia et al., 2021 [14]**	2019–2020	21	487 ± 690	7.3 ± 5.7	18.8 ± 20.5	4	17	4	8	4	3	10
**Fukuhara et al., 2021 [15]**	2011–2019	N/A	N/A	3.6 ± 0.7	N/A	N/A	N/A	N/A	N/A	N/A	N/A	N/A
**Fukuhara et al., 2021 [16]**	2011–2018	782	N/A	N/A	8.5 ± 8.9	318	77	51	168	138	211	200
**Fukuhara et al., 2021 [17]**	2019	189	N/A	N/A	5.8 ± 5.0	110	79	19	25	38	52	53
**Hirji et al., 2020 [18]**	2012–2017	227	223 ± 95	N/A	N/A	N/A	N/A	180	0	47	0	0
**Jawitz et al., 2020 [19]**	2011–2015	N/A	140.3 ± 106.6	N/A	N/A	N/A	N/A	N/A	N/A	N/A	N/A	N/A
**Malvindi et al., 2021 [20]**	N/A	13	N/A	N/A	N/A	11	2	4	0	6	0	6
**Mangner et al., 2016 [21]**	2008–2017	20	276 (150)	N/A	N/A	13	7	N/A	N/A	20	N/A	9
**Marin-Cuartas et al., 2022 [22]**	2013–2020	28	234 ± 94	5.9 ± 3.5	N/A	15	13	1	3	19	3	4
**Muensterer et al., 2022 [23]**	2008–2019	31	246.50 ± 196.3	3 ± 1.2	5.9 ± 5	18	15	6	16	16		1
**Nakazato et al., 2020 [24]**	2009–2019	4	N/A	N/A	N/A	N/A	N/A	N/A	N/A	N/A	N/A	N/A
**Tang et al., 2020 [25]**	2010–2020	269	444.75 ± 245.1	7.3 ± 8.9	N/A	137	132	55	46	115	10	61

Table reporting the main characteristics for the studies included in the meta-analysis. PVL—paravalvular leak; STS-PROM—Society of Thoracic Surgeons Predicted Risk of Mortality; SVD—structural valve deterioration; TAVR—transcatheter aortic valve replacement.

**Table 2:** Risk of bias assessment for included studies.

Study	Domain 1	Domain 2	Domain 3	Domain 4	Domain 5	Domain 6	Domain 7	Domain 8	Domain 9	Domain 10	Overall Risk of Study Bias
**Brescia et al., 2021 [14]**	No	Yes	No	Yes	Yes	Yes	No	Yes	No	Yes	**Moderate**
**Fukuhara et al., 2021 [15]**	No	Yes	No	Yes	Yes	Yes	No	Yes	No	Yes	**Moderate**
**Fukuhara et al., 2021 [16]**	No	Yes	No	Yes	Yes	Yes	No	Yes	No	Yes	**Moderate**
**Fukuhara et al., 2021 [17]**	No	Yes	No	Yes	Yes	Yes	No	Yes	No	Yes	**Moderate**
**Hirji et al., 2020 [18]**	No	Yes	No	Yes	Yes	Yes	No	Yes	No	Yes	**Moderate**
**Jawitz et al., 2020 [19]**	No	Yes	No	Yes	Yes	Yes	No	Yes	No	Yes	**Moderate**
**Malvindi et al., 2021 [20]**	No	Yes	No	Yes	Yes	Yes	No	Yes	No	Yes	**Moderate**
**Mangner et al., 2016 [21]**	No	No	No	Yes	Yes	Yes	No	Yes	No	Yes	**High**
**Marin-Cuartas et al., 2022 [22]**	No	Yes	No	Yes	Yes	Yes	No	Yes	No	Yes	**Moderate**
**Muensterer et al., 2022 [23]**	No	Yes	No	Yes	Yes	Yes	No	Yes	No	Yes	**Moderate**
**Nakazato et al., 2020 [24]**	No	Yes	No	Yes	Yes	Yes	No	Yes	No	Yes	**Moderate**
**Tang et al., 2020 [25]**	No	Yes	No	Yes	Yes	Yes	No	Yes	No	Yes	**Moderate**

**Domain 1**: Was the study’s target population a close representation of the national population in relation to relevant variables? **Domain 2**: Was the sampling frame a true or close representation of the target population? **Domain 3**: Was some form of random selection used to select the sample or was a census undertaken? **Domain 4**: Was the likelihood of nonresponse bias minimal? **Domain 5**: Were data collected directly from the subjects? **Domain 6**: Was an acceptable case definition used in the study? **Domain 7**: Was the study instrument that measured the parameter of interest shown to have reliability and validity? **Domain 8**: Was the same mode of data collection used for all subjects? **Domain 9**: Was the length of the shortest prevalence period for the parameter of interest appropriate? **Domain 10**: Were the numerators and denominators for the parameter of interest appropriate?

## Data Availability

Data pertaining to the individual publications should be sought from the study authors when appropriate.

## Supplementary Materials

The following supporting information can be downloaded at: https://www.mdpi.com/article/10.3390/jcm13051262/s1, PRISMA 2020 Checklist.
